# Methamphetamine use and correlates in two villages of the highland ethnic Karen minority in northern Thailand: a cross sectional study

**DOI:** 10.1186/1472-698X-9-11

**Published:** 2009-05-15

**Authors:** Eiko Kobori, Surasing Visrutaratna, Yuko Maeda, Siriporn Wongchai, Akiko Kada, Masako Ono-Kihara, Yoko Hayami, Masahiro Kihara

**Affiliations:** 1 Department of Global Health and Socio-Epidemiology, Kyoto University School of Public Health, Kyoto, Japan; 2 Chiang Mai Provincial Health Office, Chiang Mai, Thailand; 3 School of Human Health Science, Kyoto University Graduate School of Medicine, Kyoto, Japan; 4 Department of Clinical Research and Development, National Cardiovascular Center, Osaka, Japan; 5 Center for Southeast Asian Studies, Kyoto University, Kyoto, Japan

## Abstract

**Background:**

The prevalence of methamphetamine use and human immunodeficiency virus (HIV) incidence are high in lowland Thai society. Despite increasing social and cultural mixing among residents of highland and lowland Thai societies, however, little is known about methamphetamine use among ethnic minority villagers in the highlands.

**Methods:**

A cross-sectional survey examined Karen villagers from a developed and a less-developed village on February 24 and March 26, 2003 to evaluate the prevalence and social correlates of methamphetamine use in northern Thailand. Data were collected in face-to-face interviews using a structured questionnaire.

**Results:**

The response rate was 79.3% (n = 548). In all, 9.9% (males 17.6%, females 1.7%) of villagers reported methamphetamine use in the previous year. Methamphetamine was used mostly by males and was significantly related to primary or lower education; to ever having worked in town; to having used opium, marijuana, or heroin in the past year; and to ever having been diagnosed with a sexually transmitted infection (STI).

**Conclusion:**

Since labor migration to towns is increasingly common among ethnic minorities, the prevention of methamphetamine use and of HIV/STI infection among methamphetamine users should be prioritized to prevent HIV in this minority population in Thailand.

## Background

Historically, Thailand was once notorious for its opium production, which started in the late nineteenth century and continued until the mid twentieth century [1]. However, in modern Thailand methamphetamine is the most popular illicit drug. Of all new hospital admissions for drug treatment in Thailand in 2006, 75.6% (n = 29,235) of patients were admitted for methamphetamine use. Furthermore, 75.2% (n = 51,457) of all drug-related arrests in 2006 were methamphetamine related [2]. A household survey conducted in 2003 suggested that 0.2% of the 45 million Thai people aged 12 to 65 years had used methamphetamine during the previous year (2002), and 2.4% had used it in their lifetimes [3]. There is increasing concern that methamphetamine use is now prevalent among young people (aged 15–21 years) in Thailand. A urine test conducted among vocational school students in this age group (n = 1725) determined that 10.3% of this study group tested positive for current methamphetamine use. Additionally, 29.0% of the study group reported having ever used methamphetamine [4]. Moreover, methamphetamine use has been identified among highland ethnic minorities in areas of upper northern Thailand [5,6].

In Thailand, roughly 1 million people are members of ethnic minorities, constituting 1.6% of the entire Thai population. These minorities have distinct cultural backgrounds, practices and languages. Most (approximately 920,000) are members of nine ethnic minorities that reside in the highland areas at elevations from 500 to 2,500 meters. These highlanders are officially classified as "hill tribes," or highland ethnic minorities, among which the Karen account for the largest population (47.5%) [7,8]. Karen villagers originally resided in Myanmar for centuries but began to migrate into Thailand in the eighteenth century; today the vast majority of Karens, some 4 million, still remain in Myanmar [9]. While they face a struggle to attain their basic human rights, including democracy, and self-determination, the Karen in Thailand also face cultural and political discrimination. There is a stereotyped public view that highland ethnic minorities, including Karen residents, practice forest destruction by engaging in swidden cultivation, despite the fact that much of the deforestation has been caused by illegal logging [10]. Although the Karen have been mobile for many centuries, migration to lowland cities in search of labor or educational opportunities has increased in recent years. This was especially true in the 1980s for Karen youth. The increasing migration, together with improved infrastructure and media access in the remote villages, has resulted in a rise in material possessions that represent an elevation to prestigious cultural status as well as significant changes in lifestyle, sexual morality, and sexual behaviors [11].

Although opium is traditionally cultivated and used among some highland ethnic minorities, methamphetamine was first used in the highland communities in around 1996 [6]. Methamphetamine use was thought to be more common among Thais than among highland ethnic minorities, as reflected in the results of a recent survey of people attending a drug treatment center in northern Thailand [5]. Apart from its direct toxicity, methamphetamine represents a serious health concern in the context of the HIV epidemic. This is because methamphetamine use leads to engagement in other illicit drug use [12,13], sexual initiation or increase in sexual activity [14,15], multiple steady male partners [15], and STIs [12], though the factors associated with methamphetamine use vary depending on the study population. However, little is known about recent methamphetamine use among ethnic minority villagers in the highlands, where a rapid cultural shift is leading to increased social and cultural mixing with lowland Thai societies, in which the prevalence of methamphetamine use and HIV are high.

In 2003, we conducted a cross-sectional survey in two Karen villages, located in a mountainous area and with differing levels of development, to study the prevalence and social correlates of sexual behaviors, including drug use [16]. In this article, we reanalyze the data, focusing on the demographic and behavioral characteristics of methamphetamine users and the correlates of methamphetamine use.

## Methods

The method used in the study is described elsewhere [16]. Briefly, we conducted a survey in two Karen villages at different levels of infrastructural development in a mountainous region in northern Thailand. The two villages were selected from villages in Category 1, the most developed level, and Category 3, a less developed level, based on the government categorization; among five possible levels within that categorization, more than 90% of villages in the study districts are classified in categories 1 to 3 [7]. We recruited all 15- to 54-year-old residents for the study, assuming that the differences between villages might reflect changes in culture and consequently in the behavioral patterns of the villagers. In detail, village A had electricity and a paved road linking it to town, enabling convenient year-round access to information and town life, whereas village B had no such infrastructure, limiting the villagers' access to town, especially in the rainy season.

Data were collected on February 24 and March 26, 2003. Six Karen health workers, three for each village, conducted face-to-face interviews at the respondents' homes in each village using a structured questionnaire. The questionnaire was developed based on results of eight focus group interviews with male and female Karen villagers. The questionnaire, written in Thai, was translated into the local languages through discussions among interviewers. For sensitive questions, such as questions about drug-related and sexual attitudes or behaviors, a separate answer sheet was prepared, and illustrations were used for those who were illiterate. Prior to the data-collection phase, we pretested the questionnaire in other villages that were distant from the study villages, and then revised the questions iteratively as needed. Informed consent was obtained and no names or other identifiers were collected. After completing each interview, the consent form, questionnaire, and answer sheet were put in an envelope and sealed in front of the respondent.

For statistical analysis, the chi-square test and Fisher's exact test when necessary were used for bivariate analysis, and a multiple logistic regression analysis was used to identify variables independently associated with methamphetamine use by entering all of the variables simultaneously. *P *< 0.05 was used as the critical value to determine statistical significance. In both the bivariate and multivariable analyses, the data for males and females and the data for methamphetamine non-users (users of opium, marijuana, or heroin only) and drug non-users were combined due to the small number of females and methamphetamine non-users; this actually had a limited influence on the characteristics of the combined population. The variable "graduated from a school in town" was excluded from the multivariable analysis, since it was strongly (r > 0.7) correlated with another variable, education. Variables such as age, religion, education, and main occupation were transformed into dichotomous variables for the bivariate and multivariable analyses.

The study protocol was approved by the National Research Council of Thailand and by the Kyoto University Graduate School and Faculty of Medicine Ethics Committee.

## Results

Out of the 691 15- to 54-year-old residents of both villages, those who were not seen for three home visits or who were missing essential data on methamphetamine use, sex, age, or sexual behaviors were excluded from the analysis. This resulted in a total response rate of 79.3% (n = 548), 80.7% in village A and 76.8% in village B. None of residents visited by interviewers refused to answer the questionnaire.

Table 1 shows the situation of drug use among participants, of whom 9.9% (male 17.6%, female 1.7%) reported methamphetamine use in the past year and 13.3% (male 22.6%, female 3.5%) reported the use of at least one of four major drugs. In both villages, the drug users were predominantly male and methamphetamine was the most commonly used drug; only one participant reported injection drug use. Of the drug users, 61.0% and 36.4% of male users in Villages A and B, respectively, were multiple drug users, whereas all of the female users were single drug users.

**Table 1:** Drug use among Karen villagers in the past one year^a^

	Village A						Village B							
				
Drug use	Male (n = 174)		Female (n = 182)		Total (n = 356)		Male (n = 100)		Female (n = 92)		Total (n = 192)		Grand total (n = 548)	
				
	n	%	n	%	n	%	n	%	n	%	n	%	n	%
Methamphetamine use^c^	34	19.5	5	2.7	39	11.0	15	15.0	0	0.0	15	7.8	54	9.9
Opium use^c^	16	9.2	2	1.1	18	5.1	11	11.0	3	3.3	14	7.3	32	5.8
Marijuana use^c^	18	10.3	0	0.0	18	5.1	3	3.0	0	0.0	3	1.6	21	3.8
Heroin use^c^	11	6.3	0	0.0	11	3.1	2	2.0	0	0.0	2	1.0	13	2.4
														
Any of 4 drugs^b ^use	41	23.6	7	3.8	48	13.5	22	22.0	3	3.3	25	13.0	73	13.3
														
Methamphetamine non-use^d^	7	4.0	2	1.1	9	2.5	7	7.0	3	3.3	10	5.2	19	3.5
Non-drug use	130	74.7	174	95.6	304	85.4	74	74.0	88	95.7	162	84.4	466	85.0
Missing data	3	1.7	1	0.5	4	1.1	4	4.0	1	1.1	5	2.6	9	1.6
														
Drug injection	0	0.0	0	0.0	0	0.0	1	1.0	0	0.0	1	0.5	1	0.2

^a^Proportion of missing data varied from 0.5 to 6.7%

^b^Methamphetamine, opium, marijuana, or heroin

^c^Included multiple use

^d^Included multiple use except for methamphetamine

Table 2 describes the characteristics of the villagers according to methamphetamine use in the past year. Demographic characteristics such as age, marital status, religion, education, and graduation from a school in town were similar in both the methamphetamine users and methamphetamine/drug non-users. Methamphetamine users were more likely to be daily wage laborers, to have ever worked in town, to have used other drugs in the past year, to have ever been diagnosed with STIs in their lifetimes compared to those who were methamphetamine/drug non-users.

**Table 2:** Characteristics of villagers by status of drug use in the past one year^a^

		Methamphetamine user						Methamphetamine/drug non-user^b^					
		
Variables		Village A (n = 39)		Village B (n = 15)		Total (n = 54)		Village A (n = 317)		Village B (n = 177)		Total (n = 494)	
		
		n	(%)	n	(%)	n	(%)	n	(%)	n	(%)	n	(%)
Age group (years)	15 – 24	18	46.2	2	13.3	20	37.0	141	44.5	66	37.3	207	41.9
	25 – 34	11	28.2	3	20.0	14	25.9	85	26.8	58	32.8	143	28.9
	35 – 44	5	12.8	7	46.7	12	22.2	73	23.0	37	20.9	110	22.3
	45 – 54	5	12.8	3	20.0	8	14.8	18	5.7	16	9.0	34	6.9
													
Sex	Male	34	87.2	15	100.0	49	90.7	140	44.2	85	48.0	225	45.5
	Female	5	12.8	0	0.0	5	9.3	177	55.8	92	52.0	269	54.5
													
Marital status	Never married	17	43.6	1	6.7	18	33.3	107	33.8	57	32.2	164	33.2
													
Religion	Christianity	15	38.5	13	86.7	28	51.9	73	23.0	144	81.4	217	43.9
	Animism	3	7.7	2	13.3	5	9.3	22	6.9	9	5.1	31	6.3
	Buddhism	20	51.3	0	0.0	20	37.0	211	66.6	22	12.4	233	47.2
	Missing data	1	2.6	0	0.0	1	1.9	11	3.5	2	1.1	13	2.6
													
Education	Primary or lower	25	64.1	15	100.0	40	74.1	188	59.3	135	76.3	323	65.4
	Junior high school	7	17.9	0	0.0	7	13.0	73	23.0	21	11.9	94	19.0
	High school or higher	6	15.4	0	0.0	6	11.1	52	16.4	20	11.3	72	14.6
	Missing data	1	2.6	0	0.0	1	1.9	4	1.3	1	0.6	5	1.0
													
Main occupation	Farmer	20	51.3	15	100.0	35	64.8	215	67.8	145	81.9	360	72.9
	Daily wage laborer	14	35.9	0	0.0	14	25.9	26	8.2	10	5.6	36	7.3
	Student	0	0.0	0	0.0	0	0.0	41	12.9	20	11.3	61	12.3
	Other	4	10.3	0	0.0	4	7.4	28	8.8	1	0.6	29	5.9
	Missing data	1	2.6	0	0.0	1	1.9	7	2.2	1	0.6	8	1.6
													
Graduated from a school in town	Graduated	9	23.1	0	0.0	9	16.7	82	25.9	36	20.3	118	23.9
	Missing data	1	2.6	0	0.0	1	1.9	15	4.7	3	1.7	18	3.6
													
Ever worked in town	Ever worked	16	41.0	10	66.7	26	48.1	50	15.8	47	26.6	97	19.6
	Missing data	0	0.0	0	0.0	0	0.0	11	3.5	1	0.6	12	2.4
													
Opium use	Yes	11	28.2	6	40.0	17	31.5	7	2.2	8	4.5	15	3.0
	Missing data	0	0.0	0	0.0	0	0.0	1	0.3	1	0.6	2	0.4
													
Marijuana use	Yes	15	38.5	0	0.0	15	27.8	3	0.9	3	1.7	6	1.2
	Missing data	0	0.0	1	6.7	1	1.9	4	1.3	3	1.7	7	1.4
													
Heroin use	Yes	9	23.1	0	0.0	9	16.7	2	0.6	2	1.1	4	0.8
	Missing data	0	0.0	1	6.7	1	1.9	1	0.3	5	2.8	6	1.2
													
Opium, Marijuana, or Herion use	Yes	23	59.0	6	40.0	29	53.7	9	2.8	10	5.6	19	3.8
	Missing data	0	0.0	0	0.0	0	0.0	4	1.3	5	2.8	9	1.8
													
Ever diagnosed with STIs	Yes	5	12.8	1	6.7	6	11.1	3	0.9	3	1.7	6	1.2
	No (Ever had sex)	21	53.8	12	80.0	33	61.1	210	66.2	114	64.4	324	65.6
	No (Never had sex)	13	33.3	1	6.7	14	25.9	103	32.5	54	30.5	157	31.8
	Missing data	0	0.0	1	6.7	1	1.9	1	0.3	6	3.4	7	1.4

^a^N = 548, Those who missed the answer on methamphetamine use were excluded

^b^Including methamphetamine non-user and drug non-user

Among methamphetamine users, those from village A (developed) were more likely to be younger, to never have been married, to be daily wage laborers, to have graduated from a school in town, to have never worked in town, to have used marijuana and heroin in the past year, to have been diagnosed with an STI in their lifetimes, compared to those from Village B; no such differences were seen between villages within methamphetamine/drug non-users.

Table 3 shows the results of the bivariate and multivariable analyses of the Karen villagers. In the bivariate analysis, the respondents who were male; had never married; were not farmers; had worked in town; had used opium, marijuana, or heroin in the past year; and had been diagnosed with an STI were significantly more likely to be methamphetamine users. The multivariable analysis showed that respondents who were male; had primary or lower education; had worked in town; were opium, marijuana, or heroin users in the past year; and had ever been diagnosed with an STI were significantly more likely to be methamphetamine users.

**Table 3:** Correlates of Methamphetamine use in the past one year among Karen villagers

			MA^a ^user		Bivariate analyses			Multivariable analyses		
			
Variables		N	n	%	*P*-value	OR	95CI	*P*-value	AOR	95CI
Village	A (developed)	356	39	11.0	0.239	1.45	(0.78 – 2.71)	0.109	2.20	(0.84 – 5.78)
	B (traditional)	192	15	7.8		1.00				
										
Age group (years)	15 – 34	384	34	8.9	0.229	0.70	(0.39 – 1.26)	0.440	1.46	(0.56 – 3.82)
	35 – 54	164	20	12.2		1.00				
										
Sex	Male	274	49	17.9	0.000	11.72	(4.59 – 29.91)	0.012	3.90	(1.35 – 11.28)
	Female	274	5	1.8		1.00				
										
Marital status	Never married	182	18	9.9	0.984	1.01	(0.55 – 1.83)	0.275	2.90	(0.43 – 19.68)
	Ever married	366	36	9.8		1.00				
										
Religion	Christian	245	28	11.4	0.285	1.36	(0.77 – 2.41)	0.893	1.06	(0.44 – 2.56)
	Buddhism or Animism	289	25	8.7		1.00				
										
Education	Primary or lower	363	40	11.0	0.166	1.58	(0.82 – 3.04)	0.038	3.10	(1.06 – 9.03)
	Junior high school or higher	179	13	7.3		1.00				
										
Main occupation	Other than Farmer^c^	144	18	12.5	0.209	1.47	(0.80 – 2.69)	0.400	1.52	(0.58 – 4.01)
	Farmer	395	35	8.9		1.00				
										
Graduated from a school in town	Not graduated Graduated	402 127	44 9	10.9 7.1	0.207	1.61 1.00	(0.76 – 3.40)			-
										
Ever worked in town	Ever worked	123	26	21.1	0.000	3.69	(2.07 – 6.57)	0.003	3.55	(1.53 – 8.28)
	Never worked	413	28	6.8		1.00				
										
Opium, Marijuana or Heroin use	Yes	48	29	60.4	0.000^b^	28.45	(14.06 – 57.56)	0.000	19.63	(8.04 – 47.94)
	No	491	25	5.1		1.00				
										
Ever diagnosed with STIs	Yes	12	6	50.0	0.000	11.21	(3.19 – 39.41)	0.008	20.76	(2.18 – 197.43)
	No (Ever had sex)	357	33	9.2	0.690	1.14	(0.59 – 2.20)	0.238	2.95	(0.49 – 17.88)
	No (Never had sex)	171	14	8.2		1.00				

^a^Methamphetamine

^b^Fisher's exact test

^c^Daily wage worker (n = 50), student (n = 61), jobless (n = 26), housework (n = 6), other job (n = 1)

## Discussion

To our knowledge, this is the first study to describe the prevalence of methamphetamine use and its correlates among the Karen villagers in a mountainous area of northern Thailand. Specifically, our study revealed that in 2003 methamphetamine was readily available and was used by 9.9% of the residents of two separate Karen villages. This is a much higher rate than that reported for the general Thai population (2.4% in 2001, and 0.2% in 2003 [3]), contrary to what has been suggested in previous reports. The results presented herein strongly suggest that methamphetamine use may have spread within the Karen population since its introduction in the mid 1990s.

In contrast to our hypothesis that residential development would significantly affect the drug-use behavior patterns of the local villagers, the results of the multivariable analysis showed that experience of working in town (rather than the level of development of one's village) was the significant predictor of methamphetamine use. Contact with lowland Thai society through labor migration might have increased the use of methamphetamine because it enables laborers to work longer hours or to cope with work-related stress associated with different socio-cultural situations. It is also possible that once exposed to methamphetamine, Karen villagers might be less reluctant than Thais to use new narcotic drugs, including methamphetamine, owing to the Karen's cultural and traditional use of opium, dating from the late nineteenth century [17]. The fact that the use of opium, marijuana, or heroin was a very strong predictor of methamphetamine use supports this inference. Importantly, the multivariable analysis showed that a history of an STI was potently associated with methamphetamine use, suggesting that methamphetamine users constitute an important subpopulation of Karen villagers that should be targeted by HIV-prevention programs.

There are some limitations to our study. There may have been interviewer or reporting bias despite the intensive training of the interviewers before data collection and the use of a separate answer sheet, with illustrations for those who were illiterate, for responding to sensitive questions. The small number of methamphetamine users (n = 54) may make the model unstable and reduce the statistical power. We may not be able to generalize the results to the entire Karen population, since the results were for only two villages. Furthermore, our results may have been influenced by the so-called "war on drugs" that the Thai government started to crack down on drug businesses in February, 2003, the month our study started; however, this influence may have been mixed, since one study identified a reduction in methamphetamine use among middle school students after the "war" began [18], while another study observed a shift to methamphetamine use from injected drugs among injection-drug users [19].

## Conclusion

Despite these limitations, our study identified a high prevalence of methamphetamine use among highland Karen villagers and a strong association with experience of working in town. Since labor migration to town is increasingly common among ethnic minorities in Thailand, with the hope of achieving better economic status, the prevention of methamphetamine use and of HIV/STI infection among methamphetamine users should be given priority among minority populations in Thailand.

## Competing interests

The authors declare that they have no competing interests.

## Authors' contributions

EK planned the study and its design, carried out the data collection, analysis, and interpretation, and drafted the manuscript. SV developed the study design, coordinated the study, participated in collection the data, and made comment to the manuscript. YM participated in data analysis, data interpretation, and manuscript writing. SW was involved in coordination of the study, collection and interpretation of the data. AK was involved in data collection and analysis, and made comment to the manuscript. MOK participated in development of the questionnaire, data interpretation, and made comment to the manuscript. YH participated in development of research conception, revision of the questionnaire, and interpretation of the data from anthropological perspectives on the Karen. MK participated in planning of the study design, data analysis and interpretation, made comment to the manuscript and gave final approval for the submission of the manuscript. All authors read and approved the final manuscript.

## Pre-publication history

The pre-publication history for this paper can be accessed here:

http://www.biomedcentral.com/1472-698X/9/11/prepub
